# Commercialism in Medicine; the Pernicious Multiplying of Medical Schools*Extract from an address before the Tri-State Medical Society at Dallas, Texas, by Henry K. Leake, M. D., president, Dallas, Texas.

**Published:** 1902-01

**Authors:** 


### Abstracts and Selections.

### Commercialism in Medicine; The Pernicious Multiplying of Medical Schools.*

We are truly a commercial people, the genius of which, not always benignant, dominates our social and political life. Like an octopus it has fastened its hold upon the learned professions, particularly that of medicine, and drawn them, too, within its insatiable maw. I undervalue not the business activity of a people that expresses itself in numerous forms of intelligence and usefulness. Conventional methods of business are agreeable to society, which expects and even demands the exploiting of them in the most extravagant manner. But these methods cannot be applied in the pursuit of medicine without great detriment to its self-respect and usefulness. The wares of the tradesman and those of the medical man are of a widely different character, growing out of the peculiar education of the latter, and the conditions in which he follows his vocation. Therefore, society may encourage, or even command the medical man to advertise his mental wares as the tradesman does his wares. It may applaud and even maintain a factitious but undeserved reputation for excellence obtained in this manner, but it cannot supply genuine ability, nor by such means elevate a man in the ranks of his profession.

Society may diminish the revenue of the medical man out of all proportion to the service he renders by employing the methods of commercialism whereby his professional endowments are placed upon the block and sold to the highest bidder, and thus deprive him not only of the means of support, but of those whereby he may live the intellectual life in its fullest vigor and accomplishment. For it is not always remembered by society that the intellectual life must be cultivated by the physician more than by the members of any other learned profession. Time and opportunity must be his and withal a sufficient revenue, else this intellectual life will fail of the splendid results that, otherwise, it might attain.

Moreover, the medical profession is a silent one, its genius is not oratorical, nor self-assertive; it confirms its benefits in a firm but modest way, and well knows that its best offices are not without their limitations. These characteristics, therefore, in some degree

*Extract from an address before the Tri-State Medical Society at Dallas, Texas, by Henry K. Leake, M. D., president, Dallas, Texas.

may single out from the great mass of the profession the man of true nobility and worth, notwithstanding the upas tree of commercialism would overshadow this discerning faculty.

The evils of commercialism, in no respect, are recognized more notoriously than in the pernicious multiplying of medical schools to which at the present day social conditions invite the profession. No objection can be made to a wide diffusing of medical knowledge—

"He that directs the wandering traveler
Doth, as it were, light another's torch by his own,
Which gives him ne'er the less of light for that
it gave another."

But this knowledge, as it deals with a question of such vital importance to the community, must be of the most exalted kind. Leading medical authorities emphasize the fact that this knowledge is of such a peculiar nature that under the custom now unhappily in vogue its wide diffusion suffers as if by unwholesome attenuation, and thus loses its potency for good and augments its opportunity for harm, the very reverse of the medication to be employed against the pathology that it was designed to combat.

Within the profession, the advocates of this wide diffusing idea declare themselves the exponents of a serviceable yet unselfish philanthropy in the interest of humanity, while those among the laity enterprisingly assess the accomplishment of their designs solely upon a valuation which, perchance, may bring the profits of trade and advertisement to their respective communities. When probed to the bottom, it well may be suspected that with both classes, the idea rests upon a commercial foundation alike injurious to the welfare of the profession and even to that portion of the public itself that at least endorses or assists in its experimental application.

Opposed to this wide-diffusing idea, I by no means desire to convey the impression that I should restrict the teaching and the practice of medicine to a specially privileged class protected by the profession or by the government. Such a policy is unwise and undemocratic. But, when a wide diffusing of a medical knowledge is of an imperfect kind—the offspring of the pernicious effect of commercialism, being neither quantitatively nor qualitatively speaking within the needs of the community—then a proper regard for public safety and professional respect impels the profession, at any rate, to sound unreservedly and, if possible, to enforce a timely note of warning.

Consequently, I believe that a certain kind of concentration or centralization should be favored until an adjustment be absolutely or approximately secured between the population and physicians by limiting the opportunities for medical education, increasing the length of the medical curriculum and strengthening the latter by further amplification and exactness to fulfill the requirements of a finished and, therefore, practical medical training. The beneficent effect of this reform would be further enlarged by requiring a thorough preliminary education, or an education more closely approximating it than at present demanded. The importance of this fact was realized fully by the Greek and Roman physicians. I recall the admonition of Hippocrates. He says: "Whoever is to acquire a competent knowledge of medicine ought to possess the following advantages: a natural disposition, instruction, a favorable position for study, early tuition, love of labor, leisure." Furthermore, in his letters to his son he says: "Give due attention, my son, to geometry and arithmetic, for such studies will not only render your life illustrious and useful to your fellow beings, but your mind more acute and perspicacious in arriving at fruitful conclusions in everything pertaining to your art."

But before the student could receive the first degree of "Illumination," he must have had training in the accessory sciences; or, if this training was not complete, it must be finished with the higher philosophy of the day during the college course.

Another evil arising from this Pandora box of commercialism as affecting the multiplying of medical colleges is the social and pecuniary strife that necessarily exists between them. Students are made to have a marketable value—the lowest bid obtains them. Hence, competition in this direction is fierce, and this fact alone attracts both numbers and inferior material. In this respect the medical colleges of cities and towns are pitted against each other, likewise colleges situated in the same locality where, moreover, factional differences arise that relax the tone of the profession and discredit it with the high minded and intelligent citizen.

But the malign effects of commercialism not rarely will impress themselves upon the methods of the practitioner. Imbued with the policies of the institution from which he graduated, or else conscious of his own imperfections, or probably both, he realizes that he cannot compete with the well-trained medical man, and, consequently, he must resort to diplomacy of a low order that he may succeed pecuniarily.

This widespread evil injuriously operates upon all medical men, both trained and untrained, for necessity knows no law in the fierce struggle for existence. The highly educated medical man must in consequence lower his professional standards to the inexorable tyrant of commercialism in order to meet this unwholesome competition. He probably leads not the intellectual life with which he was inspired by his able faculty and opportunities, but money getting and maintaining a spurious reputation for superiority in common with his less informed confreres must be, henceforward, the business of his life. In reality, he thus becomes a so-called "hustler" in the widest sense of this commercial term. Certainly this is the medical drift of the times for which there seems to be no apparent remedy.

Now, the responsibility for the existence of the evils growing out of the multiplying of medical colleges must be shared by society equally with the medical profession. The chartering power of the government is administered freely and loosely. The liberties of the American citizen must not be curtailed in any direction. His is the sovereign intelligence that knows what is best for the welfare of society. His representatives in the Legislature decline to pass beneficent laws, either regulating the practice of medicine or limiting the crude and plethoric output of medical surgeons and practitioners. Were it otherwise, the hurtful desires and undertakings of the medical profession itself in these respects would be suppressed to the great advantage of both profession and society.

I have sympathy and admiration for the genius that apparently languishes for the opportunity to develop, but that rises to heights of splendid achievements despite every obstacle in its path. It bursts its prison house in all conditions that attempt to confine it. Often springing from the lower walks of life it has advanced and glorified our profession and forced its way to greater opportunities. Mr. Treves declares that we need not genius in the medical profession which is best supported by the trained, observant and plodding practitioner. But can this be true of the medical profession when genius often discovers a great flight in every other direction toward eminent usefulness? Yet genius of a high order, which is linked always with enthusiasm and intrepidity, seeks for no special laws or license in its behalf, nor is it ever suppressed finally by any adverse conditions. To restrain the people generally from the exercise of selfish instincts laws must be put in force that will bring the greatest good to the greatest number. Laws will not restrain genius—it may be left to secure its own advancement. It needs not the assistance of commercialism which might be supposed to give it the freest opportunity for its development, but which rather would misdirect and degrade its spirit and purpose.

